# Flavonoids from Bee Pollen: Functional Components, Applications, and Limitations

**DOI:** 10.3390/molecules31132379

**Published:** 2026-07-06

**Authors:** Ying Zhu, Shuting Pu, Zun Wu, Shaofang Hu, Yan Lin, Peiying Shi, Zhiguo Li, Songkun Su, Xueling Xu

**Affiliations:** 1 College of Bee Science, Fujian Agriculture and Forestry University, Fuzhou 350002, China; zhuying202402@163.com (Y.Z.); 18787333461@163.com (S.P.);; 2 College of Animal Sciences, Fujian Agriculture and Forestry University, Fuzhou 350002, China; 3 College of Biomedicine, Fujian Agriculture and Forestry University, Fuzhou 350002, China

**Keywords:** bee pollen, flavonoids, nutraceuticals, anti-inflammatory, prostatitis, colitis, neuroinflammation

## Abstract

Bee pollen is a mixture of pollen, nectar, and bee saliva. It is rich in bioactive substances, including polysaccharides, flavonoids, carbohydrates, proteins, vitamins, and minerals, conferring high nutritional and pharmacological value. The flavonoids in bee pollen are considered the main components responsible for its active function. Based on a systematic elaboration of the physicochemical properties and classification of bee pollen flavonoids, this review summarizes the preclinical research progress of bee pollen flavonoids in inflammation-related diseases, with a focus on prostatitis, colitis, and neuroinflammation, and analyzes the key bottlenecks limiting their application. It should be noted that the current evidence is primarily derived from pharmacological studies at the cellular and animal levels, and the clinical efficacy in human diseases has not yet been confirmed; their potential effects are mainly established on the basis of biological plausibility and preclinical potential. This review aims to provide a reference for subsequent fundamental research and translational exploration of bee pollen flavonoids and to offer insights for the design of future clinical trials.

## 1. Introduction

Pollen, the male reproductive cell of plants produced by the stamens [1], is spread to the pistil by wind, insects, or other means to complete pollination. Bee pollen is an irregularly shaped pollen mass collected by bees from the stamens of flowering plants, mixed with nectar and bee saliva [2]. Based on the pollen-collecting behavior of bees, various types of pollen traps are placed at the hive entrance to artificially intercept the pollen pellets collected by bees [3]. After collection, the pollen undergoes removal of impurities, drying, and sterilization and is then transformed into edible bee pollen products. The types of bee pollen are mainly distinguished based on the plant source from which bees collect, with common types including rapeseed bee pollen, corn pollen, sunflower bee pollen, locust flower bee pollen, and buckwheat bee pollen. Among these, rapeseed pollen is the most common in China, characterized by its yellow color, distinctive fresh scent, and rich aroma.

Bee pollen is an important dietary supplement, hailed as a treasure trove for human nutrition, and is gradually being used as a functional food [4]. This is mainly due to its rich content of nutrients such as polysaccharides, proteins, unsaturated fatty acids, vitamins, flavonoids, and trace elements [5,6]. Additionally, the nutritional composition of bee pollen is influenced by various factors, including plant species, geographical location, soil origin, and season of the year in which the pollen is collected [7,8]. The extraction methods used during the collection of bee pollen can also affect its nutrient concentration and biological activity [9]. Research has shown that these factors lead to variations in the composition and pharmacological actions of bee pollen [10].

Bee pollen is considered a potential source of natural antioxidants, capable of counteracting the oxidative stress that underlies the pathogenesis of many diseases, primarily due to the presence of flavonoids [11]. Flavonoids in bee pollen are a class of secondary metabolites formed during the growth process of plants [12]. As important physiologically active substances, they comprise over a dozen types of glycosides and their aglycones, exhibiting excellent health-preserving and medicinal effects. Denisow et al. [13] have shown that the flavonoids and other polyphenolic compounds in bee pollen exhibit antioxidant activity.

In recent years, with increasing research on the therapeutic potential of natural products, the roles of flavonoids from bee pollen in the intervention of inflammatory diseases have attracted growing attention. Previous studies have documented the antioxidant and anti-inflammatory bioactivities of bee pollen flavonoids. However, systematic summaries are still lacking regarding their action mechanisms in specific disease models, standardized analytical characterization methods, and technical bottlenecks in practical applications.

This review focuses primarily on prostatitis, colitis, and neuroinflammation for the following reasons. Although these conditions belong to the urogenital, digestive, and nervous systems, respectively, they share an inflammation and oxidative stress imbalance as a common pathological node. The antioxidant and anti-inflammatory activities of bee pollen flavonoids provide a biological basis for their potential intervention in these diseases. Meanwhile, existing experimental studies have accumulated sufficient evidence in these three areas to support cross-comparison and mechanistic synthesis. Furthermore, the current lack of satisfactory therapeutic options for these diseases further highlights the research value of such natural product-based intervention strategies. On these grounds, this review systematically collates and integrates the relevant literature.

A review of the existing literature reveals that Rzepecka-Stojko et al. [11] focused on the structure–activity relationship of bee pollen polyphenols concerning their chemical structure, absorption, metabolism, and antioxidant activity. However, their review did not include in-depth mechanistic analyses of specific disease models or cover application-related aspects such as extraction methods and wall disruption techniques. Denisow and Denisow-Pietrzyk [13] evaluated the biological and therapeutic properties of bee pollen from a holistic nutritional perspective, primarily positioning it as a dietary supplement. Their review lacked a dedicated analysis of flavonoid compounds and did not systematically address specific inflammatory diseases such as prostatitis, colitis, or neuroinflammation. More recently, Qiao et al. [14] published a review focusing on nutritional components and wall disruption techniques, providing a systematic compilation of phenolamides and flavonoid glycosides. Nevertheless, the core focus of their work was not on flavonoids, and aspects such as drying processes and allergenic risks were not covered.

Based on the above research gaps and the rationale for the topic selection, this review systematically summarizes the physicochemical properties, extraction methods, and analytical and characterization techniques of bee pollen flavonoids and provides an in-depth exposition of their preclinical intervention effects on the three aforementioned inflammatory diseases. This review explicitly distinguishes three levels of research evidence, namely whole bee pollen, crude extracts, and isolated monomeric compounds, while critically discussing the key bottlenecks limiting their application, including drying processes and allergenic risks. This comprehensive perspective is intended to provide a theoretical reference for the integration of preclinical research on bee pollen flavonoids and their translational development directions, as well as to offer insights for the design of subsequent clinical trials.

## 2. Chemical Characteristics of Flavonoids in Bee Pollen

### 2.1. Structural Classification and Representative Compounds of Flavonoids

**Figure 1:**
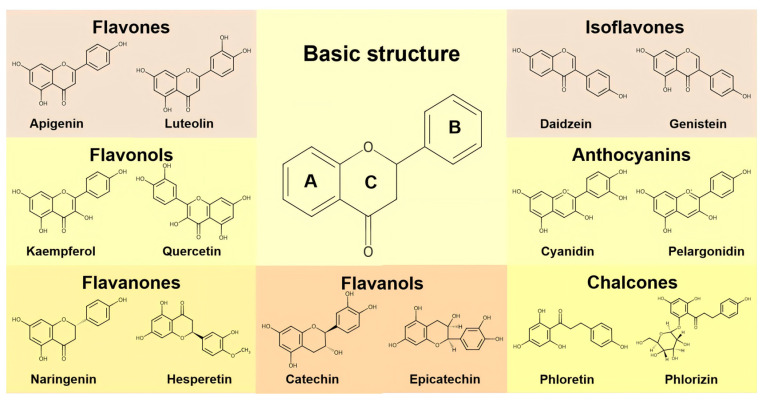
The basic structure of flavonoids and their classes.

Flavonoids are secondary metabolites with a C6-C3-C6 skeleton. They are divided into multiple subclasses according to the oxidation degree, substitution sites and cyclic structure of the C-ring (Figure 1) [12,15].

Flavones constitute an important subclass of flavonoids. They represent the simplest flavonoid structures with a 2-phenylchromone as the parent nucleus. A double bond exists between the C-2 and C-3 positions of the C-ring. A ketone group is located at the C-4 position of the C-ring. Flavones are widely distributed in leaves, flowers and fruits. They mainly occur in the form of glycosides. Apigenin and luteolin belong to this subclass.

Flavonols are structurally derived from flavones. They possess an additional hydroxyl group at the C-3 position of the C-ring [16]. Flavonols are the most ubiquitous flavonoids in foods. They are widely found in various fruits and vegetables. Kaempferol, quercetin, myricetin and rutin are the most extensively investigated flavonols.

Isoflavones are structural isomers of flavones. The B-ring is attached to the C-3 position of the C-ring. They exhibit estrogenic activity in certain animal models and are generally regarded as phytoestrogens. Isoflavones show a limited distribution in the plant kingdom and mainly exist in leguminous plants. Genistein and daidzein are their typical representatives.

Flavanones have a saturated bond between C-2 and C-3 of the C-ring. A carbonyl group is present at the C-4 position. These compounds commonly coexist with corresponding flavones and flavonols in plants. The saturated bond between C-2 and C-3 of the C-ring is the key structural difference between flavanones and other flavonoid subgroups. Flavanones are prevalent in all citrus fruits, such as oranges and lemons. Hesperetin and naringenin are their typical representatives.

Flavanols contain a single bond between C-2 and C-3. A hydroxyl group rather than a carbonyl group is attached at the C-3 position. They generally exist as monomers, such as catechins and polymers such as proanthocyanidins. Flavanols are widely distributed in tea, cacao and grape seeds. Catechin and epicatechin are their representative compounds.

Chalcones are flavonoids with an open-chain structure. They lack the typical tricyclic skeleton. Chalcones often act as precursors for other flavonoids [17]. Phlorizin and phloretin are their typical representatives.

Anthocyanins are glycosidic forms of anthocyanidins. They are also water-soluble pigments. These compounds mainly accumulate in the outer cell layers of various fruits and regulate plant coloration [18], such as the hues of flowers, fruits and autumn leaves. The most widely studied anthocyanins include cyanidin, pelargonidin, petunidin and peonidin.

### 2.2. Composition and Content Variations in Flavonoids in Bee Pollen

A variety of flavonoids have been identified in bee pollen, including epicatechin, catechin, and phloretin [14]. Flavonoids are essential for pollen tube formation and fertilization in flowering plants [19]. As bioactive secondary metabolites, flavonoids determine the flavor, color and pharmacological activities of pollen. Most flavonoids in bee pollen exist in the form of glycosides [11]. Glycosylation increases the polarity of flavonoids. It helps prevent cytoplasmic damage and enables their stable storage in cellular vacuoles.

Total flavonoid contents vary considerably among different bee pollen samples. De-Melo et al. [20] reported values ranging from 0.3 to 19.0 mg QE/g (quercetin equivalents) in Brazilian monofloral bee pollen of various botanical origins, whereas Oroian et al. [21] documented a range of 4.90 to 20.45 mg QE/g (quercetin equivalents) in 24 samples from Romania. Collectively, the total flavonoid content of bee pollen generally falls within the range of 0.3–20.45 mg QE/g, with considerable variations depending on botanical origin and geographical location.

The concentrations of individual flavonoids also show remarkable differences. Thakur and Nanda [22] determined the contents of major flavonoids in Indian bee pollen. The values were 3.14–15.94 mg/100 g (0.0314–0.1594 mg/g) for quercetin, 0.12–9.35 mg/100 g (0.0012–0.0935 mg/g) for kaempferol, 4.81–24.83 mg/100 g (0.0481–0.2483 mg/g) for rutin, and 1.06–5.86 mg/100 g (0.0106–0.0586 mg/g) for luteolin. The variations in these contents are mainly associated with plant origin, geographical location and harvesting season.

Flavonoid profiles in bee pollen are markedly affected by botanical origin and geographical conditions. Rape bee pollen from China contains bioactive components including kaempferol, isorhamnetin and quercetin [23]. Brazilian Melipona subnitida bee pollen is rich in isorhamnetin, naringin and naringenin [24]. Naringenin and rutin are not detected in bee pollen from Cistus sp. of Spain [25]. Plant growth status and metabolic activity vary with seasons. Such changes further regulate flavonoid synthesis. Seasonal variation in temperature and light conditions influences the biosynthesis of different flavonoid subclasses, thereby altering the types and contents of flavonoids in bee pollen.

### 2.3. Analytical and Characterization Methods for Flavonoids

The analysis and characterization of flavonoids in bee pollen consist of qualitative identification and quantitative determination. Current studies show a tendency to rely on conventional analytical methods. Most studies only conduct routine quantification via HPLC-DAD. Sufficient attention is not paid to the in-depth characterization of unknown components. This section systematically reviews relevant methods, including conventional chromatographic separation, structural confirmation using high-resolution mass spectrometry and systematic data mining strategies. It also highlights the limitations and misconceptions of each method in practical application.

#### 2.3.1. High-Performance Liquid Chromatography with Diode Array Detector (HPLC-DAD)

HPLC-DAD is the most widely adopted method for routine quantitative analysis of flavonoids in bee pollen. Its popularity mainly stems from its simplicity and low cost rather than technical superiority. This technique achieves separation based on partition differences between the stationary phase and mobile phase. Detection is performed using ultraviolet-visible absorption spectra. Flavonoids exhibit characteristic absorption peaks at 240–280 nm and 300–380 nm. These peaks can support preliminary structural identification. Isomers usually present highly similar ultraviolet spectra. DAD alone cannot distinguish such compounds.

This method has an essential limitation. Its qualitative analysis heavily relies on comparison with reference standards. Commercial standards are unavailable for most trace flavonoid glycosides and isomers in bee pollen. A large number of potential active ingredients are thus systematically overlooked. Bakour et al. [26] analyzed pollen samples from 14 different plant species by HPLC-DAD. Their identification results were limited to compounds with available reference standards. This method cannot detect new components. Accordingly, HPLC-DAD is suitable for routine quality control of known constituents. It is not recommended as the sole approach for exploratory research.

#### 2.3.2. Ultra-High-Performance Liquid Chromatography Coupled with Diode Array Detector and Tandem Mass Spectrometry (UHPLC-DAD-MS/MS)

UHPLC-DAD-MS/MS is an important tool for the qualitative identification of flavonoids in bee pollen, with its technical advantages arising from the high separation capacity of ultra-high-performance liquid chromatography, the UV spectral acquisition capability of the diode array detector, and the structural characterization power of tandem mass spectrometry. It should be noted, however, that the analytical scope of UHPLC-MS/MS is not inherently confined to targeted modes; it can be operated in targeted, untargeted, or pseudo-targeted acquisition modes, depending on the experimental design and acquisition method. In targeted mode, the identification results are constrained by predefined compound lists and database coverage, whereas in untargeted mode, the combination with high-resolution mass spectrometry enables structural elucidation of unknown compounds.

Using this method, Thakur and Nanda [22] identified 60 polyphenolic compounds in bee pollen, comprising 38 flavonoids and their derivatives, 21 phenolic acids and their derivatives, and one glucosinolate, among which daidzin and sinigrin were reported for the first time in bee pollen. These identification results were also limited by the predefined compound lists and database coverage. In summary, UHPLC-DAD-MS/MS is suitable for the multi-dimensional verification of known compounds when operated in targeted acquisition mode, but it should not be used as a standalone approach for untargeted screening or the discovery of novel compounds; the latter requires untargeted acquisition strategies coupled with high-resolution mass spectrometry.

#### 2.3.3. Ultra-High-Performance Liquid Chromatography-Electrospray Ionization-Quadrupole Time-of-Flight Mass Spectrometry (UHPLC-ESI-QTOF-MS)

UHPLC-ESI-QTOF-MS integrates the high separation efficiency of ultra-high-performance liquid chromatography, the efficient ionization capacity of an electrospray ionization source, and the high-resolution mass measurement capability of quadrupole time-of-flight mass spectrometry. It can provide accurate molecular weights with errors below 5 ppm and high-resolution secondary fragment ions. This technique acts as a core tool for untargeted screening and identification of unknown flavonoids in bee pollen. Compared with conventional liquid chromatography, UHPLC uses columns packed with sub-2 μm particles. It achieves a higher separation degree and resolution within a shorter analysis time. The ESI source enables soft ionization of polar flavonoids and effectively retains molecular ion information. Unlike low-resolution mass spectrometry, QTOF-MS can deduce molecular formulas and speculate chemical structures without reference standards. It is particularly valuable for identifying flavonoid glycosides that lack commercial standards.

Nevertheless, its technical drawbacks cannot be ignored. Firstly, qualitative results obtained by this method are speculative. Accurate molecular weights and fragment information fail to confirm the linkage positions of sugar chains, sugar configurations and hydroxyl substitution patterns. Such fine structural details require verification by NMR. Secondly, identification relies heavily on database coverage. Numerous flavonoid glycosides in bee pollen are not included in public databases, which may lead to misidentification or undetected components.

Rocchetti et al. [27] used UHPLC-ESI-QTOF-MS to annotate 467 compounds in Italian bee pollen. They screened 35 phenolic compounds as markers for distinguishing botanical origins, among which flavonoids were the predominant group. The annotations were obtained through database matching. The fine structures were not further validated. Therefore, these results should be regarded as tentative annotations rather than definitive identifications. UHPLC-ESI-QTOF-MS is suitable for untargeted screening, chemotaxonomic marker screening and preliminary structural deduction of candidate compounds. Nuclear magnetic resonance spectroscopy is still required for the confirmation of new compounds and reference standards.

#### 2.3.4. Nuclear Magnetic Resonance Spectroscopy (NMR)

NMR is the gold standard for structural confirmation of flavonoids, and it is particularly effective in determining the linkage positions, configurations and sequences of sugar moieties in glycosides. Two-dimensional nuclear magnetic resonance techniques such as HMBC can provide long-range hydrogen–carbon correlation information and act as a reliable approach for distinguishing the linkage sites of sugar chains. Nevertheless, the limitations of this technique in practical applications are often underestimated. It presents low sensitivity and requires milligram-level pure samples. Two-dimensional NMR experiments usually take several hours to several days. In addition, sample preparation is complicated, as individual monomers need to be isolated and purified from complex mixtures.

Li et al. [28] combined LC-DAD-ESI-MS and NMR with a stepwise separation strategy to identify the structures of four flavonoid glycosides from rape bee pollen. High-purity samples were prepared via semi-preparative HPLC for subsequent structural confirmation by NMR. This workflow is only feasible for components that can be obtained in sufficient pure quantity. It indicates that currently identified flavonoid structures in bee pollen represent merely the tip of the iceberg. A large number of trace components have long been overlooked due to technical constraints. Accordingly, NMR is suitable for accurate structural verification of key markers, but it cannot support high-throughput analysis.

#### 2.3.5. Chemometrics and Multivariate Statistical Analysis

Chemometric methods including principal component analysis, hierarchical cluster analysis, linear discriminant analysis and artificial neural networks perform dimensionality reduction on high-dimensional data acquired by chromatography or mass spectrometry. They uncover intrinsic differences between bee pollen samples of distinct origins. The improper application of these methods requires caution. Multivariate statistical models can distinguish any two sample groups effortlessly. Distinctions may stem from noise or batch effects. Uncritical acceptance of model outcomes may result in the identification of false-positive markers.

Thakur and Nanda [22] applied UHPLC-DAD-MS/MS combined with multivariate statistical analysis and artificial neural networks to screen Indian bee pollen based on antioxidant properties and polyphenol composition. Nevertheless, external validation of the models, namely the predictive performance on independent samples, was not adequately reported in this study. The robustness of its conclusions remains to be further verified. Moreover, the differential compounds screened out by chemometrics still require structural confirmation via tandem mass spectrometry or nuclear magnetic resonance spectroscopy. Without such confirmation, these variables are only statistically significant rather than authentic chemical markers. Accordingly, chemometrics serves as an effective tool for discovering potential markers. Its results must undergo dual validation, including chemical verification and biological verification.

#### 2.3.6. Critical Recommendations for Method Selection

Based on the above analysis, the analysis and characterization of flavonoids in bee pollen cannot rely on a single all-purpose technique. Different methods should be rationally combined according to research objectives. Most existing studies mainly adopt HPLC-DAD or UHPLC-DAD-MS/MS for routine analysis. The exploration of trace components and new compounds is insufficient.

A hierarchical strategy is recommended for future research. UHPLC-ESI-QTOF-MS is first used for untargeted screening to obtain candidate differential compounds. Sufficient pure substances are then acquired via preparative separation. Nuclear magnetic resonance spectroscopy is finally applied for structural confirmation. This strategy is particularly suitable for in-depth mining of key markers. Only in this way can the current plateau in the research on flavonoid constituents of bee pollen be broken, a plateau marked by repeated reports of known compounds and a lack of progress in identifying new chemical structures.

The effective implementation of the above tiered strategy relies on the systematic collation and comparison of existing research data. Table 1 summarizes the analytical results of De-Melo et al. [20] on monofloral bee pollen from five different botanical origins in Brazil, intuitively demonstrating the significant impact of botanical origin on the flavonoid composition and content of bee pollen.

### 2.4. Chemotaxonomic Significance and Quality Control Markers

Flavonoids have significant value in the chemotaxonomy of bee pollen. Pollen from different botanical families and genera exhibit characteristic flavonoid profiles, which can be used to distinguish the botanical origin of bee pollen [29]. For example, bee pollen from Brassica (rapeseed) is rich in characteristic flavonoids, particularly glycosides of quercetin and kaempferol. Qiao et al. [30] further analyzed the phenolamide and flavonoid glycoside profiles of 20 types of monofloral bee pollen and clearly proposed that characteristic compounds can serve as chemical markers for botanical origin identification. This taxon-specific distribution of flavonoids not only reflects the botanical origin of bee pollen but also provides a rational foundation for developing origin-specific quality control markers.

In terms of quality control markers, total flavonoid content is the most commonly used indicator, but its insufficient specificity makes it difficult to use alone for botanical origin identification. More promising markers include characteristic flavonoid glycosides, such as quercetin-3-O-β-D-glucosyl-(2→1)-β-glucoside and kaempferol-3, 4′-di-O-β-D-glucoside identified from rapeseed bee pollen by Li et al. [28], as well as flavonoid ratios, such as the kaempferol-to-quercetin ratio. Thakur and Nanda [22] analyzed the polyphenolic composition of Indian bee pollen from different origins, and their data showed variations in flavonoid composition among different sources, suggesting that flavonoid ratios may serve as auxiliary identification indicators. Future research is recommended to establish a standardized fingerprint library for bee pollen flavonoids, integrating HPLC fingerprint similarity evaluation, quantification of characteristic flavonoid glycosides, and flavonoid ratio analysis to achieve rapid and reliable botanical origin identification and quality evaluation.

## 3. Wall Disruption of Bee Pollen and Extraction of Flavonoids

The choice of extraction method directly affects the separation efficiency and identification accuracy of flavonoids in bee pollen. Unlike common plant matrices, bee pollen has a hard outer wall composed of sporopollenin, which poses a significant barrier to conventional solvent extraction. Therefore, the extraction of flavonoids from bee pollen must consider both wall disruption treatment and solvent selection as two core issues.

### 3.1. Wall Disruption Methods for Bee Pollen

The complex cell wall structure of bee pollen is a major obstacle limiting the release and absorption of its nutrients. The pollen wall consists of two layers: the inner layer is composed of cellulose and pectin, while the outer layer is rich in sporopollenin [31]. Although this complex structure effectively protects the intracellular nutrients, it also makes it difficult for the human digestive system to completely break it down, thereby limiting the bioavailability of the nutrients [32]. Therefore, breaking the cell wall is a key prerequisite for improving the digestibility and absorption rate of bee pollen nutrients and for fully realizing its functional activities.

Currently, the commonly used wall disruption methods include physical methods (such as ultrasonic disruption and high shear disruption), chemical methods (acid or alkali treatment), and biological methods (such as enzymatic hydrolysis). It should be noted that no single wall disruption method can simultaneously meet the requirements of high efficiency, low cost, and good flavonoid stability. Although physical methods are the most widely used, ultrasound primarily disrupts the exine through cavitation effects, and the heat generated by high shear treatment can easily lead to the degradation of heat-sensitive flavonoids. Chemical methods have high efficiency, but the risk of reagent residue is prominent, which may damage the flavonoid structure and affect safety. Biological methods have mild conditions but relatively low efficiency and carry a risk of microbial contamination.

Given the limitations of single-wall disruption techniques, combined wall disruption strategies have become an effective approach to overcome the above shortcomings. Studies have shown that protamex hydrolysis can degrade the exine and disintegrate the intine at the germinal apertures. Ultrasound treatment can break the exine into fragments, but its effect on the intine is limited. In contrast, the combined treatment of protamex hydrolysis and ultrasound can synergistically disrupt both the exine and the intine, achieving a more thorough wall disruption effect [33].

### 3.2. Physicochemical Properties of Bee Pollen Flavonoids and Their Extraction Adaptability

Flavonoids generally appear as gray-yellow, orange-yellow, or light-yellow solid powders at room temperature, with their color originating from the absorption of specific wavelengths of light by their conjugated aromatic system. Most flavonoids have poor water solubility, but their water solubility can be improved by forming glycosidic bonds with sugars, as seen in rutin. Due to the presence of multiple hydroxyl groups and other polar functional groups, flavonoids are generally polar and exhibit high solubility in polar organic solvents such as methanol, ethanol, and acetone [34]. In contrast, nonpolar or weakly polar solvents result in low extraction efficiency. Therefore, polar solvents or water–organic solvent mixtures are the preferred choices for extracting flavonoids from bee pollen. In addition, some flavonoids are heat-sensitive and prone to degradation at high temperatures. It is recommended to use low-temperature techniques such as ultrasound-assisted extraction or to strictly control the temperature during conventional extraction.

### 3.3. Solvent Selection and Extraction Efficiency

Solvent polarity is a key factor affecting the extraction efficiency of flavonoids from bee pollen. Lawag et al. [35] showed that the use of 70% ethanol in water maximized the extraction yield of phenolic acids and flavonoids from unground bee pollen. However, the extraction efficiency varies significantly among different flavonoid classes: 35% to 90% ethanol in water is suitable for most flavonoids, while low-polarity flavonoid aglycones require high concentrations of ethanol or ethyl acetate. This difference suggests that using a single solvent cannot achieve efficient extraction of all flavonoids, and solvent ratios should be optimized according to the target compounds.

In addition, the pH of the extraction medium also significantly affects the extraction efficiency of flavonoids. Available studies indicate that acidic conditions generally favor higher extraction yields of most flavonoids. The mechanism is that a low pH suppresses the ionization of phenolic hydroxyl groups, maintaining flavonoids in their neutral, non-ionized form, which enhances their hydrophobicity and solubility in organic solvents and thereby improves extraction efficiency [34]. Therefore, for the extraction of flavonoids from bee pollen, it is recommended to maintain the pH of the extraction medium in the acidic range to optimize extraction performance.

### 3.4. Comparison of Different Extraction Methods

Current extraction methods for flavonoids from bee pollen mainly include solvent extraction, microwave-assisted extraction, ultrasound-assisted extraction, and enzyme-assisted extraction [34]. The mechanisms and limitations of these four methods are compared in Table 2.

Among these methods, ultrasound-assisted extraction is one of the most widely used techniques for extracting flavonoids from bee pollen. Compared with traditional solvent extraction, it offers advantages such as shorter extraction time, lower energy consumption, and the ability to minimize degradation of heat-sensitive components. However, the extraction efficiency of this technique is not optimal under all conditions. For example, for unground whole bee pollen, conventional agitation extraction can be more effective than ultrasound-assisted extraction [35]. Therefore, the selection of the extraction method should comprehensively consider sample characteristics and the physicochemical properties of target compounds rather than blindly pursuing advanced technology.

### 3.5. Emerging Green Extraction Technologies

Given the environmental burden of traditional organic solvents, various green extraction technologies have emerged in recent years. Deep eutectic solvents offer advantages such as high designability, low toxicity, good biodegradability, and high extraction efficiency, showing application potential for the extraction of flavonoids from bee pollen. Subcritical water extraction utilizes the tunable polarity of water under high temperature and pressure, enabling flavonoid extraction without organic solvents. However, the high temperatures involved may compromise the stability of thermolabile flavonoids. Pulsed electric field-assisted extraction improves extraction efficiency by inducing electroporation of cell membranes, consumes low energy, and is suitable for heat-sensitive components.

It should be noted that the application of the above green technologies in bee pollen is still in its early stages, and systematic evaluations of extraction efficiency, cost effectiveness, and scalability are lacking. Most current studies remain at the laboratory level and are still far from industrial application.

## 4. The Role of Bee Pollen Flavonoids in Inflammatory Diseases

Bee pollen, as a natural and multifunctional health supplement, exhibits potential in the intervention of various diseases due to its rich nutritional components and bioactive substances. Its potential benefits can be partially attributed to the abundant flavonoids it contains, which possess antioxidant, anti-inflammatory, and immunomodulatory biological activities. When the body encounters pathogens, allergens, or tissue injury, the inflammatory response is activated as a physiological defense mechanism. However, persistent inflammation may lead to metabolic disorders and contribute to the development of various chronic diseases. In this process, key enzymes, including cyclooxygenases and protein kinases, along with pro-inflammatory cytokines such as IL-1, IL-6, and IL-8, collectively drive the inflammatory cascade, ultimately resulting in tissue damage.

Bee pollen flavonoids have demonstrated a variety of potential pharmacological activities in preclinical studies. They are able to alleviate oxidative stress and inhibit the production of inflammatory mediators such as cytokines and chemokines, showing potential to mitigate inflammation-related pathological changes in animal models of chronic non-bacterial prostatitis (CNBP), colitis, and neuroinflammation. In addition, these compounds can modulate immune responses and may promote the repair and regeneration of damaged tissues. However, the above findings are mainly derived from experimental studies at the cellular and animal levels, and their clinical efficacy remains to be further validated. Overall, bee pollen flavonoids exhibit intervention potential based on biological plausibility in the aforementioned inflammatory diseases, but their clinical application still requires more research to be confirmed.

### 4.1. Preclinical Evidence for Bee Pollen Flavonoids in Chronic Non-Bacterial Prostatitis

Prostatitis is an inflammation of prostate tissue caused by various complex factors [40]. CNBP, also known as chronic pelvic pain syndrome, is the most common type of prostatitis, accounting for approximately 90% of clinical cases [41]. Patients often experience persistent pain in the lower abdomen, perineum, or lower back, accompanied by symptoms such as dysuria, frequent urination, and urgency, which severely affect their quality of life [42]. Current clinical treatment mainly relies on α-blockers, nonsteroidal anti-inflammatory drugs, and antibiotics [43]. Although these medications can alleviate some symptoms, they are associated with long treatment duration, significant toxic side effects, poor prognosis, and a high recurrence rate. Therefore, exploring safer and more effective therapeutic approaches has emerged as an urgent clinical need.

**Figure 2:**
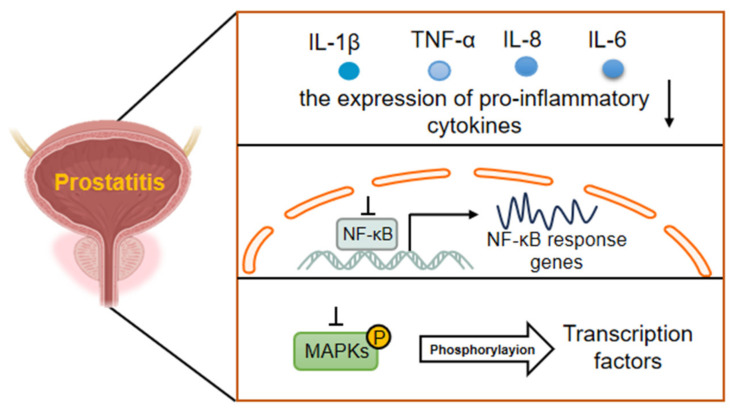
The figure illustrates the key inflammatory signaling pathways involved in the pathogenesis of prostatitis. It highlights the central role of elevated pro-inflammatory cytokines, including IL-1β, TNF-α, IL-8, and IL-6. These cytokines activate two major signaling pathways: the NF-κB pathway, which induces the expression of NF-κB-responsive genes, and the MAPK pathway, characterized by the phosphorylation of kinases and subsequent activation of transcription factors. Together, these interconnected pathways sustain a chronic inflammatory state, driving tissue damage and disease progression in prostatitis.

Experimental studies on wall-disrupted whole bee pollen have demonstrated that rape pollen represents an effective natural source for alleviating symptoms associated with CNBP. Qiao et al. [44] found that whole bee pollen, without component isolation or purification, could improve relevant indicators in CNBP models through selective modulation of the gut microbiota, with the effect being particularly pronounced at a high dose (1.26 g/kg) of cell wall-disrupted pollen. Experiments using a carrageenan-induced rat CNBP model showed that high-dose cell wall-disrupted rape pollen reduced prostate wet weight by approximately 32%, decreased the prostate index by 36%, and significantly suppressed the expression of IL-6, IL-8, IL-1β, and TNF-α (Figure 2). It should be emphasized, however, that the aforementioned effects may arise from the synergistic actions of multiple components in pollen—including flavonoids, phenolic acids, polysaccharides, and proteins—and cannot be attributed solely to flavonoids.

Studies based on isolated flavonoids provide more direct evidence for the anti-inflammatory activity of flavonoids (Table 3). Naringin can reduce prostaglandin E2 production, inhibit COX-2 expression, and block NF-κB activation. Quercetin reduces the expression of inflammatory factors by inhibiting the NF-κB and MAPK signaling pathways [45]. However, current mechanistic studies exhibit a tendency toward homogenization, with most work stopping at the detection of common indicators such as NF-κB, MAPK, and inflammatory cytokines, resulting in a notable lack of in-depth analysis of the structure–activity relationships of different flavonoid monomers. For example, the differences between naringin and quercetin in terms of NF κB inhibition potency, upstream regulatory molecules, and downstream effector gene selectivity have not been systematically investigated.

In addition, the pharmacokinetic properties, prostate distribution, and metabolic characteristics of flavonoids from non-bee pollen plant sources, including puerarin, luteolin, kaempferol, and pinocembrin, have been preliminarily investigated in animal studies [46]. Using the aerial parts of *Glycyrrhiza uralensis* Fisch. as the test material, that study demonstrated that the aforementioned flavonoids were widely distributed in the prostate, and their metabolites were also detectable in prostate tissue, suggesting that flavonoids may exert local effects within the prostate. Notably, kaempferol is one of the major aglycones of flavonoid glycosides in bee pollen [14]. This structural commonality provides indirect support for the biological plausibility of bee pollen flavonoids in the intervention of CNBP. However, given the marked differences in glycosylation patterns and specific glycoside compositions between licorice flavonoids and bee pollen flavonoids, the above pharmacokinetic data cannot be directly extrapolated to the in vivo behavioral characteristics of bee pollen-derived flavonoids. Nevertheless, the oral bioavailability of flavonoids is generally low, mainly attributable to their poor water solubility, extensive intestinal metabolism, and efflux transporter-mediated elimination. To date, direct studies on the actual accumulation concentrations, major active metabolite forms, and transmembrane transport mechanisms of bee pollen flavonoids in prostate tissue remain very limited. This constitutes not only a core limitation of current research on bee pollen flavonoids but also a major pharmacological bottleneck that hinders their translation from laboratory research to preclinical applications.

**Table 3:** Preclinical evidence for flavonoids in chronic non-bacterial prostatitis.

Test Material	Source	Material Type	Level of Evidence	Experimental Models	Dosage	Main Findings	Reference
Quercetin	Commercial	Isolated compound	Animal	Chronic prostatitis model induced with complete Freund’s adjuvant in Sprague Dawley rats	Oral administration of quercetin for 4 weeks	Decreased the expression of pro-inflammatory cytokines IL-1β, IL-2, IL-6, IL-17A, and TNFα, improved antioxidant capacity, and inhibited the phosphorylation of NF-κB and MAPKs	[45]
Prosta-Q (commercial combination supplement containing quercetin, bromelain, and papain)	Commercial	Commercial combination supplement	Observational human	Prostatic secretions samples from 70 patients and 8 asymptomatic controls	500 mg quercetin (as Prosta-Q), twice daily for 4 weeks	Decreased oxidative stress metabolites and increased endorphin levels in prostatic secretions	[47]
Kaempferol	Commercial	Isolated compound	In vitro	LPS-induced prostate organoid inflammation model	20, 40, and 80 μM kaempferol were co-treated in each well for 24 h	Reduced inflammatory cytokine expression; activated the Nrf2 antioxidant pathway; decreased mitochondrial ROS production, thereby alleviating mitochondrial damage in LPS-induced prostate organoids	[48]

### 4.2. Preclinical Evidence for Bee Pollen Flavonoids in Colitis

Colitis is an inflammatory disease, with major types including inflammatory bowel diseases such as ulcerative colitis and Crohn’s disease [49]. Patients typically present with abdominal pain, diarrhea, and fever, with bloody stools, weight loss and other complications in severe cases [50]. The compound annual growth rate of colitis incidence in China has reached 9.4%, and the pathogenesis involves complex interactions among genetic factors, environmental exposures, gut microbiota dysbiosis, and immune responses [51]. Commonly used clinical drugs such as mesalazine and corticosteroids have side effects including nausea, vomiting, and headache. Surgical intervention can also lead to infection, bleeding, and malabsorption of nutrients. Therefore, the development of safe and effective therapeutic strategies is urgently needed.

**Figure 3:**
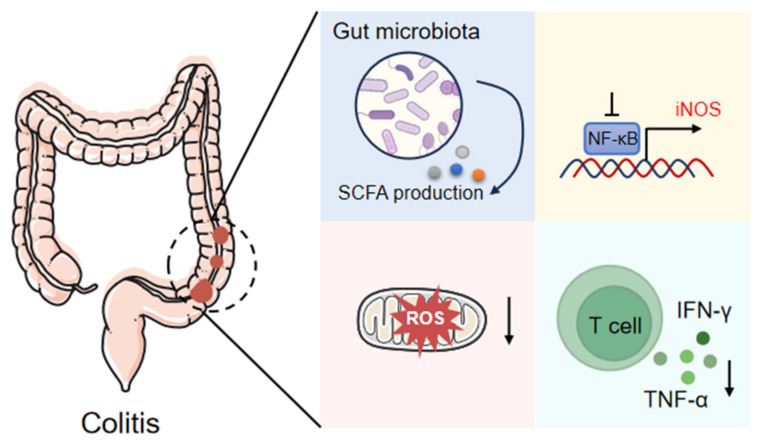
This schematic diagram illustrates the proposed mechanistic pathway through which gut microbiota dysbiosis contributes to the pathogenesis of colitis. Dysbiosis leads to impaired production of short-chain fatty acids (SCFAs), which in turn promotes the activation of pro-inflammatory pathways, including iNOS and NF-κB. These events collectively drive the development of colitis and further stimulate T cell activation, resulting in the release of key inflammatory cytokines such as IFN-γ and TNF-α. This cascade perpetuates a cycle of inflammation and tissue damage characteristic of the disease.

Experimental studies on crude extracts of whole bee pollen have demonstrated that Schisandra bee pollen exerts significant protective effects in colitis models. Cheng et al. [52] found that administration of Schisandra bee pollen extract at a dose of 20.4 g/kg significantly improved dextran sodium sulfate-induced colitis in mice, as evidenced by restoration of colonic tissue structure and epithelial barrier integrity, a reduction in oxidative stress, and a decrease in inflammation levels (Figure 3). Gut microbiota analysis revealed that the extract remodeled the microbial balance, increased diversity, and enriched beneficial bacteria associated with short-chain fatty acid production and immune regulation, such as Akkermansia and Lactobacillus. It should be noted that the observed ameliorative effects may result from the synergistic action of multiple components in the crude extract and cannot directly prove that flavonoids are the sole active components.

Studies based on isolated and purified flavonoid monomers provide more direct evidence (Table 4). In a dextran sodium sulfate-induced rat model of colitis, oral administration of quercetin (1–5 mg/kg/day) promoted the repair of inflamed intestinal mucosa, and its mechanism was associated with downregulation of the NF-κB pathway and reduction in iNOS expression [53]. Lv et al. [54] found that hyaluronic acid nanoparticles loaded with apigenin-Mn(II) increased antioxidant enzyme content and significantly improved damaged colonic tissue. In addition, quercetin reduced the production of IFN-γ and TNF-α in T lymphocytes stimulated with concanavalin A, exerting an anti-inflammatory effect to alleviate intestinal inflammation [55].

It is noteworthy that existing studies have focused heavily on common pathways such as NF-κB, MAPK, and inflammatory cytokines (TNF-α, IL-6, IFN-γ), leading to a severe lack of exploration into the differences in the effects of different flavonoid monomers. For example, although quercetin and apigenin belong to the same class of flavonoids, their similarities and differences in regulating NF-κB signaling intensity, upstream activating molecules, and target gene selectivity have not yet been systematically analyzed. Furthermore, all current relevant studies are limited to animal models, and no randomized controlled clinical trials have been reported. The translation from animal experiments to clinical applications faces the following challenges: a dose conversion dilemma, as the doses of flavonoid monomers used in animal studies (e.g., quercetin 1–5 mg/kg/day) differ by one to two orders of magnitude from the actual content of these monomers in bee pollen; and a lack of safety data, as toxicological data from long-term use and the risks of drug interactions remain unclear.

### 4.3. Preclinical Evidence for Bee Pollen Flavonoids in Neuroinflammatory Diseases

Neuroinflammation is a fundamental immune response of the central nervous system to various stimuli. It is characterized by the release of inflammatory mediators from activated microglia and astrocytes, as well as the recruitment of innate and adaptive immune cells to the site of inflammation. This process inhibits neuronal differentiation, induces apoptosis and degenerative changes, and consequently leads to various neurodegenerative diseases. Neuroinflammation can increase the permeability of the blood–brain barrier, allowing peripheral inflammatory mediators to infiltrate the central nervous system and further exacerbate inflammation. Therefore, inhibiting neuroinflammation is considered a key strategy for the treatment of neurodegenerative diseases.

**Figure 4:**
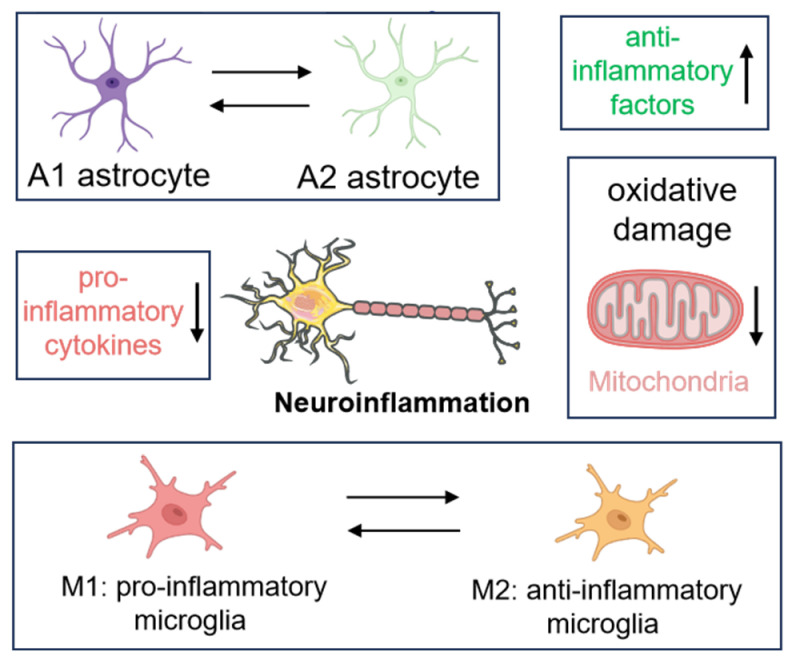
This schematic diagram illustrates the cellular and molecular mechanisms underlying neuroinflammation, focusing on the dual roles and dynamic interactions of astrocytes and microglia. The central process of neuroinflammation is driven by an imbalance between pro-inflammatory and anti-inflammatory cellular states. Microglia polarize into the classical M1 (pro-inflammatory) phenotype, which releases pro-inflammatory cytokines, or the alternative M2 (anti-inflammatory) phenotype, associated with anti-inflammatory factors. Similarly, astrocytes adopt either a detrimental A1 state or a protective A2 state in response to these signals. The sustained release of pro-inflammatory cytokines contributes to oxidative damage and mitochondrial dysfunction, forming a vicious cycle that perpetuates neuroinflammation and leads to neuronal injury.

Preliminary evidence from whole bee pollen studies. Aabed et al. [61] investigated the effects of bee pollen on neuroinflammation in a rodent model of autism. The experiment included a control group, a model group (propionic acid 250 mg/kg for 3 days), a bee pollen treatment group (250 mg/kg for 4 weeks), and a propolis treatment group (250 mg/kg for 4 weeks). The results showed that the neurotoxic effect of propionic acid was manifested as a significant increase in IL-6 and a significant decrease in IL-10. Both bee pollen and propolis effectively alleviated the neurotoxic effects of propionic acid, as the IL-6 and IL-10 levels in the treatment groups were not significantly different from those in the healthy control group (Figure 4). It should be noted that this study used whole bee pollen without isolation or purification, and the observed neuroprotective effects cannot be attributed solely to flavonoid compounds, as they may result from the synergistic action of multiple components.

Studies based on isolated and purified flavonoid monomers provide more direct evidence for their modulatory effects (Table 5). In vitro experiments showed that rutin could upregulate PP2A levels to regulate tau protein hyperphosphorylation and downregulate the NF-κB pathway, thereby inhibiting glial cell activation and improving cognitive dysfunction [62]. In vivo studies confirmed that quercetin inhibited astrocyte activity and reduced inflammatory factor levels by modulating the Sirtuin1/NLRP3 pathway [63]. Apigenin ameliorated behavioral deficits and nigral dopaminergic system degeneration in a rat model of Parkinson’s disease and inhibited NF-κB activation to prevent neuroinflammation and oxidative stress [64]. Naringenin enhanced the antioxidant defense system composed of SOD, CAT, and GSH and reduced TNF-α and IL-6 levels [65].

A prominent issue is that the aforementioned studies have repeatedly focused on common pathways, such as NF-κB, NLRP3, TNF-α, IL-6, and IFN-γ, while lacking in-depth investigation into the differential mechanisms of action of various monomers such as rutin, quercetin, apigenin, and naringenin. Furthermore, most studies have used high-concentration interventions in vitro. Whether these flavonoid monomers can still act on the same targets at physiologically relevant concentrations in vivo remains to be verified.

Unlike other inflammatory diseases, the treatment of neuroinflammation faces the unique obstacle of blood–brain barrier penetration. The oral bioavailability of flavonoids is generally low, and their target exposure in the central nervous system may be far below the effective concentrations used in in vitro experiments. This pharmacological bottleneck has rarely been addressed in existing studies. In addition, the differences among rutin, quercetin, apigenin, and naringenin in terms of NF-κB inhibition potency and upstream regulatory molecules have not been systematically compared. Currently, all related studies are limited to in vitro experiments or animal models, and no clinical trial has been reported.

## 5. The Limitations of Bee Pollen Application

Bee pollen possesses rich pharmacological activities and has beneficial effects on human health, making it a functional food. Although bee pollen shows promise in preclinical interventions for various diseases, there are also some limitations in its application that may hinder its further development and clinical translation.

### 5.1. Effects of Drying Methods on Bee Pollen Quality

Fresh bee pollen has a water content of 20% to 30%. This high water activity can easily lead to caking, mildew, and microbial contamination, while also causing oxidation of unsaturated fatty acids and degradation of heat-sensitive components such as vitamin C and flavonoids, significantly affecting product quality [70]. Therefore, an appropriate drying process is key to ensuring bee pollen quality.

Currently, the main drying methods for bee pollen include hot air drying, freeze drying, vacuum pulse drying, and microwave drying. These methods differ significantly in cost and quality retention (Table 6).

Hot air drying (including sun drying) is the most commonly used method. Song et al. [76] showed that hot air drying at 40 °C maximally preserved the nutrients of lotus bee pollen. Freeze drying avoids high-temperature oxidation through ice crystal sublimation. Estevinho et al. [77] compared the effects of hot air drying and freeze drying on bee pollen quality and found that the indicators of freeze-dried samples were significantly better than those of hot air-dried samples. However, the high equipment investment and energy consumption limit its large-scale application. Vacuum pulse drying can significantly improve efficiency and reduce energy consumption, but Wang et al. [74] pointed out that when the temperature exceeds 55 °C, hydroxymethylfurfural (HMF) and α-dicarbonyl compounds increase significantly, and the loss rate of seven essential amino acids exceeds 32.2%. Although microwave drying is fast and energy-saving, Conte et al. [78] showed that microwave treatment may reduce the content of antioxidant compounds in bee pollen, and its uneven heating characteristics may also cause local overheating, leading to the Maillard reaction and degradation of active components.

The choice of drying method should comprehensively consider production scale, cost, and quality requirements. Small enterprises or individual users may prefer hot air drying, while large-scale industrial production may adopt freeze drying, vacuum pulse drying, or microwave drying. Regardless of the method chosen, the key is to ensure that the water content of the dried bee pollen is appropriate to maintain the best quality and nutritional value.

### 5.2. Allergic Reactions Caused by Bee Pollen

Bee pollen is a natural cocktail of floral pollen, nectar, and secretions produced by honeybees and has the potential to cause allergic reactions in humans [79]. Allergic reactions caused by bee pollen are most commonly Type I allergies [80]. The mechanism of a Type I allergic reaction involves the allergen stimulating B cells to differentiate into plasma cells that produce allergen-specific IgE antibodies. These IgE antibodies then attach to the surface of mast cells or basophils, placing the body in a sensitized state. When the body encounters the same allergen again, the allergen binds to the IgE attached to the mast cells or basophils, activating these cells and triggering an allergic reaction [81]. Symptoms of an allergic reaction generally include skin itching, sneezing, difficulty breathing, throat swelling, and so on. Notably, in addition to plant pollen, the salivary gland secretions and other components contained in bee pollen may also act as allergenic factors, and the diverse sources of bee foraging further increase the complexity of allergy risks.

Enzymatic treatment is an effective method for degrading allergens in plant-based foods [82]. Through enzymatic hydrolysis of bee pollen by using specific enzymes, such as glucozyme, cellulase and papain, the structure of allergenic proteins in bee pollen can be broken down or altered, thereby reducing their reactivity with the human immune system. At the same time, enzymatic treatment can also be applied to pollen wall rupture to promote the release of nutrients and bioactive components in bee pollen. Tao and colleagues explored the effects of combined treatment with cellulase, pectinase, and papain on allergenic proteins in *Brassica campestris* bee pollen [83]. Five potential allergens were identified in the samples, including pre-fibrin, cystatin, alcohol dehydrogenase, gliadin, and expansin. After enzymatic hydrolysis of Brassica rapa var. pekinensis bee pollen using a complex of pectinase, cellulase, and papain, the content of three potential allergenic proteins (pre-fibrin, cystatin, and alcohol dehydrogenase) was significantly reduced, and the content of oligopeptides and amino acids in the main components was significantly increased. Immune blotting experiments also confirmed that the binding ability of bee pollen proteins to specific immunoglobulin antibodies was significantly reduced after enzymatic treatment, thereby reducing the allergenicity of bee pollen.

Even though bee pollen appears to be safe and is sometimes recommended by doctors for the prevention and treatment of certain diseases, it should be avoided by certain groups of people. For instance, individuals with liver disease, honey intolerance, or allergies to other bee products should not consume bee pollen. Before using bee pollen and its extracts for disease prevention and treatment, further experimental and clinical studies on their bioactive components should be conducted to better evaluate their safety and efficacy. As bee pollen becomes more widely promoted and used in the future, addressing the sensitization issues caused by bee pollen should be given top priority. This will ensure that the benefits of bee pollen can be safely realized while minimizing risks associated with its use.

### 5.3. Other Important Limitations of Bee Pollen Application

In addition to processing techniques and allergenicity, the widespread application of bee pollen as a functional food or drug candidate faces the following key challenges.

#### 5.3.1. Natural Variability of Chemical Composition

The chemical composition of bee pollen is strictly regulated by its botanical origin. Bee pollen produced by plants of different families and genera, such as Brassica of *Brassicaceae*, Robinia of *Fabaceae*, and Citrus of *Rutaceae*, exhibits significant differences in the composition of flavonoids, phenolic acids, polysaccharides, and amino acids. These differences make it difficult to maintain consistent bioactivity across different batches of products. Although this natural variability reflects the diversity of bee pollen, it poses serious challenges to standardized production, quality control, and the reproducibility of clinical studies. Establishing a chemical composition database for bee pollen based on botanical origin and clarifying the range of variation for each source is a prerequisite for achieving product uniformity.

#### 5.3.2. Effects of Geographical Origin and Harvest Season

Geographical origin and harvest season are two additional key factors affecting the chemical quality of bee pollen. Climatic conditions (temperature, humidity, light), soil types, and environmental pollution levels in different regions can cause significant fluctuations in the types and contents of flavonoids in bee pollen. Furthermore, plant growth cycles and metabolic rhythms vary by season. Bee pollen harvested in spring and summer is generally superior to autumn samples in terms of antioxidant activity and active component profiles. Currently, systematic comparative studies on bee pollen from different geographical origins and harvest seasons remain scarce. The lack of standardized harvest protocols and quality grading systems has led to inconsistent quality of commercially available products.

#### 5.3.3. Risk of Pesticide Residues

During pollen collection, bees may come into contact with pesticides applied in farmland, orchards, and forest environments, leading to residues of agrochemicals such as organophosphates, pyrethroids, and neonicotinoids in bee pollen. Pesticide residues not only reduce the food safety of bee pollen but may also pose risks to human health through long-term low-dose exposure, including neurotoxicity, endocrine disruption, and reproductive toxicity. Currently, systematic monitoring data on pesticide residues in bee pollen are very limited, and relevant international and domestic maximum residue limits remain inadequate. There is an urgent need to establish high-throughput detection methods and safety limit standards covering multiple classes of pesticides.

#### 5.3.4. Accumulation of Heavy Metals and Environmental Contaminants

As a biological indicator of environmental exposure, bee pollen has a strong ability to accumulate heavy metals [84]. Heavy metals such as lead, cadmium, mercury, and arsenic mainly originate from industrial emissions, traffic exhaust, mining activities, and agricultural inputs. These metals can be absorbed by plant roots and transferred to pollen. Long-term intake of bee pollen with excessive heavy metal content may cause chronic damage to the kidneys, liver, and nervous system. Currently, systematic risk assessment of heavy metal contamination in bee pollen and unified standards for maximum residue limits have not been established, limiting its safe consumption as a functional food.

#### 5.3.5. Inadequate Quality Control System

Given the variability and contamination factors described above, the standardization of bee pollen products faces multiple obstacles. Currently, the quality control of commercially available bee pollen mainly relies on general indicators such as total flavonoid content and total phenolic content. However, these indicators show significant overlap among different botanical origins and lack sufficient specificity, making it difficult to effectively distinguish product sources or verify product authenticity. Furthermore, the absence of characteristic chemical markers such as flavonoid glycosides and phenolamides, along with their quantitative standards, results in a lack of comparability among products from different batches or origins. This situation severely hinders the upgrade of bee pollen from a natural raw material to a standardized functional product.

## 6. Conclusions

As humans increasingly pursue a balanced and healthy diet, bee pollen has gradually become a focus of attention in the fields of food and dietary supplements due to its rich nutritional composition and health benefits. Bee pollen is not only an indispensable nutritional source for honeybees, but its bioactive components, including proteins, vitamins, flavonoids, minerals, and essential amino acids, also exert multiple biological effects on the human body, such as enhancing immunity, as well as exerting antioxidant and anti-inflammatory activities.

Flavonoids are important active substances in bee pollen. Current studies at the cellular and animal levels have demonstrated their antioxidant and anti-inflammatory activities. Although these lines of evidence remain at the preclinical stage, they provide biological plausibility for exploring the intervention potential of bee pollen flavonoids in inflammation-related diseases, including chronic prostatitis, colitis, and neuroinflammation. However, their clinical efficacy and safety still require validation through well-designed clinical trials. In addition, the chemical composition of bee pollen is influenced by factors such as botanical origin, geographical environment, and harvesting season, leading to variations in flavonoid composition among different batches, which may affect the reliability and reproducibility of experimental results. Therefore, the establishment of standardized production processes and quality control systems is a critical prerequisite for ensuring the comparability of research findings on bee pollen and its active components. Meanwhile, current techniques for cell wall disruption and drying of bee pollen still have certain limitations, and future efforts should be directed toward improving the release efficiency of nutrients while maximizing the retention of their bioactivity.

In summary, bee pollen flavonoids demonstrate intervention potential based on biological plausibility in inflammation-related diseases, but the current evidence has not yet reached the stage of supporting clinical application. Future research should, on the basis of standardized preparation, further explore their molecular mechanisms of action, conduct systematic in vivo pharmacodynamic and toxicological evaluations, and promote well-designed clinical trials so as to provide a more solid scientific foundation for the medical application of bee pollen flavonoids.

## Figures and Tables

**Table 1:** Total flavonoid content, identified flavonoids, and bioactivities of monofloral bee pollen from different botanical origins.

Bee Pollen	Botanical Origin	Geographic Origin	Extraction Method	Analytical Platform	Identified Flavonoids	Total Flavonoid Content (TFC)	Reported Bioactivity	References
Monofloral bee pollen	*Cocos nucifera*	Una, Bahia, Brazil	Solvent extraction method	HPLC-MS	Isorhamnetin rutinoside, rutin, and others	1.1 ± 0.0 mg QE/g	Antioxidant, antimicrobial	[20]
Monofloral bee pollen	*Alternanthera*	Ribeirão Preto, São Paulo, Brazil	Solvent extraction method	HPLC-MS	Catechin, rutin, and others	1.0 ± 0.1 mg QE/g	Antioxidant, antimicrobial	[20]
Monofloral bee pollen	*Anadenanthera*	Ribeirão Preto, São Paulo, Brazil	Solvent extraction method	HPLC-MS	Catechin, ampeloptin, rutin, and others	1.3 ± 0.1 mg QE/g	Antioxidant, antimicrobial	[20]
Monofloral bee pollen	*Mimosa caesalpiniaefolia*	Neópolis, Sergipe, Brazil	Solvent extraction method	HPLC-MS	Catechin, rutin, quercetin, and others	1.3 ± 0.0 mg QE/g	Antioxidant, antimicrobial	[20]
Monofloral bee pollen	*Myrcia*	Arvorezinha, Rio Grande do Sul, Brazil	Solvent extraction method	HPLC-MS	Quercetin diglucoside, rutin, myricetin, and others	19.0 ± 0.6 mg QE/g	Antioxidant, antimicrobial	[20]

**Table note:** QE, quercetin equivalents. Solvent extraction refers to the conventional extraction method. HPLC-MS, high-performance liquid chromatography-mass spectrometry. “And others” indicates that additional flavonoid compounds were identified but not fully listed in the summarized data. Values are expressed as mean ± standard deviation.

**Table 2:** Comparison of common extraction methods for flavonoids.

Extraction Method	Principle	Advantages	Disadvantages	Reference
Solvent extraction method	Solubility of flavonoids varies with solvent	The operation is simple; the cost is low	Long-time and high solvent consumption required	[36]
Microwave-assisted extraction	The microwave can penetrate the solvent and selectively heat the target component, which in turn produces a thermal effect that causes the flavonoids contained in the plant to dissolve in the extract	Microwave heating is fast, which can greatly reduce the extraction time and make more efficient use of energy	The cost of the equipment is high, and special security measures are required	[37]
Ultrasound-assisted extraction	Ultrasonic-assisted solvents and enzymes are used to extract flavonoids	Extraction time is short, the efficiency is high, and it is gentle on heat-sensitive compounds	In large-scale applications, it can be difficult to guarantee uniformity of processing	[38]
Enzymatic digestion	Enzymes are used to disrupt the cellulose-based cell wall structure and the pectin connected between cells so that the pectin in the plant is completely decomposed into small molecules, and the mass transfer resistance of extraction is reduced	High selectivity and increased extraction rate for specific compounds	Enzyme selection and condition optimization are complex	[39]

**Table 4:** Preclinical evidence for flavonoids in colitis.

Test Material	Source	Material Type	Level of Evidence	Experimental Models	Dosage	Main Findings	Reference
Naringenin	Commercial	Isolated compound	Animal	Dextran sulfate sodium induced colitis in male BALB/c mice	Diet with 0.3% naringenin for 9 days	Decrease disease activity index, increase colon length, and inhibit inflammatory cytokines such as IL-6 and IL-17A	[56]
Quercetin-loaded microcapsules	Prepared	Isolated compound	Animal	Acetic acid induced colitis in mice	Oral administration of quercetin microcapsules (quercetin microcapsules are prepared using pectin/casein polymers)	Neutrophil recruitment in the colon was reduced, histological alterations were mitigated, the production of the inflammatory cytokines IL-1β and IL-33 was reduced, and the anti-inflammatory cytokine IL-10 was prevented	[57]
Quercetin	Commercial	Isolated compound	Animal	Adoptive T cell transfer model of chronic colitis	Quercetin was orally administered at a dose of 10 mg/kg body weight for 7 weeks	Reduce the inflammatory response in the colon, decrease the expression of pro-inflammatory cytokines such as IFN-γ and TNF-α, and increase the expression of anti-inflammatory cytokines	[58]
Kaempferol	Commercial	Isolated compound	Animal	Dextran sulfate sodium induced colitis in mice	Administer kaempferol (50 mg/kg/day, dissolved in 1% carboxymethyl cellulose sodium) by gavage for 14 days	Alleviate the gross symptoms of dextran sulfate sodium induced colitis in mice and alleviate colonic injury, upregulation of IL-10 transcription and downregulation of the expression of inflammation-associated genes	[59]
Kaempferol	Commercial	Isolated compound	In vitro	Epithelial–endothelial cells coculture model; lipopolysaccharides induce intestinal inflammation and barrier dysfunction	80 µM kaempferol	Inhibit the NF-κB signaling pathway activation, ameliorate the lipopolysaccharide-induced decrease in protein expression of zonula occludens-1, occludin, and claudin-2	[60]

**Table 5:** Preclinical evidence for flavonoids in neuroinflammatory diseases.

Test Material	Source	Material Type	Level of Evidence	Experimental Models	Dosage	Main Findings	Reference
Luteolin	Commercial	Isolated compound	Animal + In vitro	Autologous blood was injected into rats to establish the intracerebral hemorrhage model in vivo, and oxyhemoglobin was used to mimic the intracerebral hemorrhage model in vitro	The different groups were injected with 5, 10, and 20 mg/kg luteolin	Inhibit microglia activation, prevent the activation of the TLR4/TRAF6/NF-κB signaling pathway	[66]
Luteolin	Commercial	Isolated compound	Animal	A rat model of subarachnoid hemorrhage	Different groups were treated with 10, 30, 60 and 90 mg/kg luteolin	Inhibit subarachnoid hemorrhage-induced neuroinflammation, reduce activation of microglia and infiltration of neutrophils, decrease release of pro-inflammator	[67]
Quercetin	Commercial	Isolated compound	Animal	Lipopolysaccharides induce anxiety-like behaviors and neuroinflammation in rats	Daily administration of quercetin (10, 50, and 100 mg/kg) for 21 days	Improve lipopolysaccharide-induced anxiety-like behavior, reduce inflammatory marker levels in the brain, increase brain-derived neurotrophic factor mRNA levels, and decrease inducible nitric oxide synthase mRNA levels	[68]
Kaempferol	Commercial	Isolated compound	Animal	Lipopolysaccharides induce striatal damage in mice	Intraperitoneal injection of kaempferol 50 mg/kg pretreatment for 7 days	Alleviate lipopolysaccharide-induced damage to striatal neurons, inhibit the activation of microglia, and inhibit the production of pro-inflammatory cytokines and chemokines	[69]

**Table 6:** Comparison of common drying methods for bee pollen.

Drying Method	Principle	Advantages	Disadvantages	Reference
Dry naturally	The heat of the sun is used to evaporate water from bee pollen.	Simple and easy, low cost, not limited by equipment	The drying process is limited by weather conditions, and the quality of the product is unstable	[71]
Hot air drying	Bee pollen is dried with hot air to promote the transfer of heat and mass from bee pollen and remove the water in it	The equipment is simple, easy to operate, and the temperature is controllable	The energy consumption is high, and the bee pollen is easy to oxidize during the drying process, resulting in the loss of color and nutrients	[72]
Freeze drying	The water-containing bee pollen is pre-frozen to below the freezing point to form ice crystals, which are then directly sublimated into gas in a vacuum environment to achieve drying	Dried bee pollen is as high as possible and retains the original nutrients	The production cost is high, the drying time is long, and the equipment requirements are high	[73]
Vacuum pulse drying	In a vacuum environment, the boiling point of water is reduced, and the evaporation of water is accelerated to achieve the purpose of drying	Inhibits the oxidation reaction of the material and can better preserve the nutrients in the pollen	The cost of equipment is higher, the technology is more complex, and it is difficult to popularize	[74]
Microwave drying	The electromagnetic waves generated by the microwave generator interact with the substance to create a thermal effect, which heats up quickly and removes moisture	The drying speed is fast, the heating is uniform, energy is saved, and it is easy to automatically control	Uneven microwave energy distribution can lead to localized overheating, which in some cases can affect product quality	[75]

## Data Availability

Data will be made available on request.
